# Phytochemical Profiling and Biological Evaluation of *Anthyllis henoniana* Organs: Integrated In Vitro and Molecular Docking Insights Into Antioxidant, Anti‐Inflammatory, and Cytotoxic Potential

**DOI:** 10.1002/cbdv.202503501

**Published:** 2026-08-06

**Authors:** Sabrine Jaber, Rami Rahmani, Jalloul Bouajila, Mohamed Debouba

**Affiliations:** ^1^ Laboratoire De Recherche “Biodiversité, Molécules et Applications LR22ES02'', Institut supérieur De Biologie Appliquée Université De Gabés Gabés Tunisia; ^2^ Become: Technology, Science, AI & Automation Lab Paris France; ^3^ Laboratoire De Génie Chimique Université De Toulouse, CNRS, INPT Toulouse UT France

**Keywords:** *Anthyllis henoniana*, bioactivity, in silico analysis, in vitro analysis

## Abstract

*Anthyllis henoniana* leaves and flowers were subjected to fractionated extraction using cyclohexane (CYHA), dichloromethane (DCM), ethyl acetate (EtOAc) and methanol (MeOH). The leaves‐MeOH extract showed the highest yield and total polyphenol contents (TPC) compared to the flowers. The strongest antiradical activity (IC_50_ = 7.7 µg/mL) was recorded for the leaves compared to flowers (>50 µg/mL). The HPLC‐DAD analysis showed the presence of eight and twelve bioactive compounds in the leaf and flower extracts, respectively. The flowers‐CYHA extract exhibited the best anti‐5‐lipoxygenase (anti‐5‐LOX) activity (IC_50_ = 15 µg/mL) compared to the leaves (IC_50_ = 42 to 47 µg/mL). *Anthyllis henoniana* leaves showed greater anti‐acetylcholinesterase (anti‐AChE) activity than the flowers. The leaves‐DCM fraction induced 60 and 70% cell growth inhibition against MCF‐7 and HCT‐116, respectively. Furthermore, the molecular docking investigations supported the in vitro antioxidant and anti‐5‐LOX patterns, revealing that the identified compounds exhibited low binding energies and interacted effectively within the novel human peroxidase (PDB:1PRX) and the crystal structure of soybean lipoxygenase‐B (PDB: 2IUJ) enzymes, respectively. The obtained results indicated that the leaves‐MeOH extract was the most enriched in polar compounds with high antioxidant and cytotoxic capacities, whereas the flowers‐CYHA extracts accumulated nonpolar compounds of an effective anti‐inflammatory capacity.

## Graphical abstract

*Anthyllis henoniana* aerial parts were subjected to fractionated extraction. Methanolic fractions yielded high total phenolic and flavonoid contents with strong antiradical (anti‐DPPH) and neuroprotective (anti‐AChE) activities, whereas dichloromethane and cyclohexane fractions demonstrated potent cytotoxicity (anti‐MCF‐7, anti‐HCT‐116) and anti‐inflammatory (anti‐5‐LOX) potential, respectively. HPLC‐DAD identified 8 leaf and 12 flower metabolites, supported by in silico docking into 1PRX and 2IUJ target proteins.

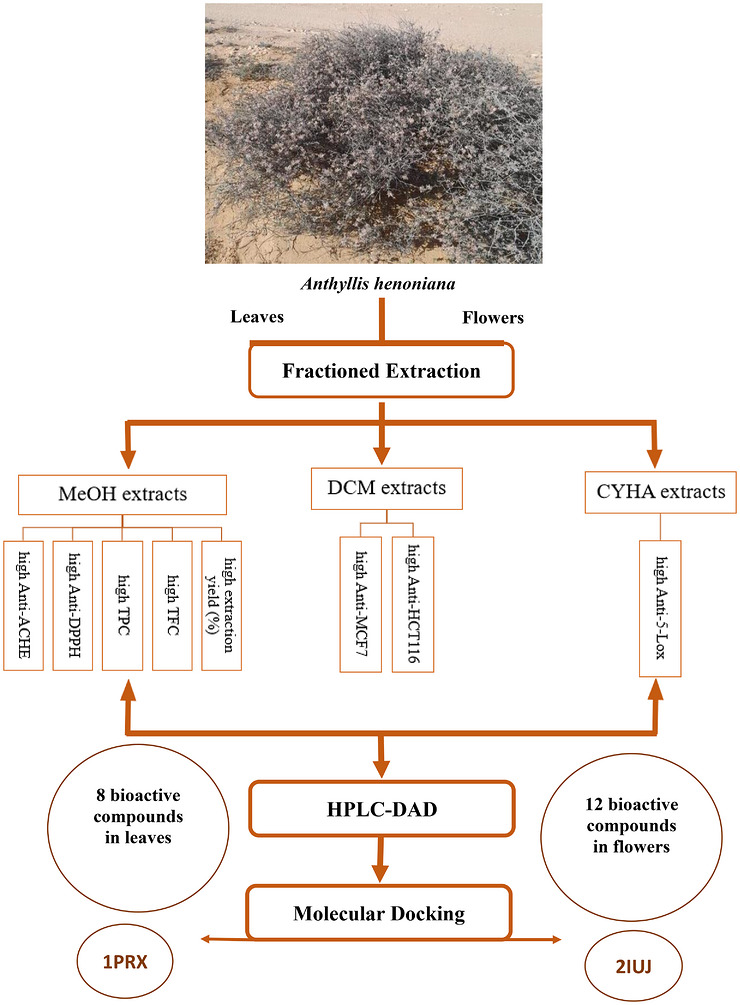

## 1 Introduction

Among the 2162 plant species wide speared in the rangelands of Tunisia [1], about 135 are specific to pre‐saharian lands [2]. Gamoun et al. [3]. assessed five predominant plant families in these areas including Asteraceae, Poaceae, Brassicaceae, Chenopodiaceae and Fabaceae, which account for about 50% of the Tunisian desert flora. Pre‐Saharan lands are essentially steppes formed by chamaephytes and annual species. These arid lands are often settled by the *Anthyllis* genus steppes growing on limestone crusts topped with soil or sandy loam [2]. The genus *Anthyllis*, which belongs to the Fabaceae family, includes a large number of flowering plants that are mainly distributed in Europe, the Mediterranean Basin, and North Africa [4]. This genus includes about 20 to 25 species, grouped into herbaceous, shrubby, or perennial. Among the *Anthyllis* genus, *A. henoniana* is an endemic specie of North Africa (Tunisia, Algeria, and Libya) belonging to Fabaceae family, subfamily Papilionoideae [5]. It is exclusively encountered in the extreme south regions of Tunisia (Beni khedeche, Dhaher, ouara), where it has a pastoral value, namely for camels and goats and sometimes for sheep [6]. Besides its pastoral value, *A. henoniana* roots improve soil fertility in such marginal lands by hosting a diversity of nitrogen‐fixing rhizobium strains [7, 8].

*A. henoniana* is a leguminous chamaephyte with a canopy that is 40 to 50 cm high and a yellowish‐white flowers inflorescence cluster that appears in April. *A. henoniana* seeds display high germination capacity under optimal conditions and it has evolved morphological and anatomical adaption to favor the interception and absorption of dew or rain by shoots rather than roots under water deficit conditions [9]. Moreover, the adaption of xerophytes species, such as *A. henoniana*, to pre‐Saharan climate involves metabolic and physiological strategies to overcome water deprivation and high temperatures. It was shown that a high secondary metabolites and polyamines levels enables water deficit adjustment and protects plant cells against external biotic and abiotic constraints [10]. Drought arising from water lack and high temperature was found to increase flavonoids and phenolics [11], anthocyanins, and glycosides in higher plants [12]. Plant species widely spread in arid and infra‐arid conditions usually build up a chemical defense against any external stressful factors [13]. Many studies have shown that extreme climate conditions induced the secondary metabolites production in plant tissues [14]. It was established that plant secondary metabolites play an important role in the growth, development, reproduction and plants defense against abiotic stresses such as high light intensity, low temperatures, UV‐B radiation, heavy metals, and nutrient deficiencies [15]. Moreover, these secondary compounds are involved in plant protection against pathogens and predators, and also exhibit other important properties such as antimicrobial and antioxidant activity [16].

Moreover, it was shown that secondary metabolites contribute to plant interaction with the extreme environment conditions [17], and are sources of natural products of high pharmaceuticals and nutraceuticals proprieties [18].

Recent literature has increasingly focused on the therapeutic potential of *Anthyllis henoniana*, with studies illustrating the biochemical profile, antioxidant capacity, and both the antidiabetic and antihyperlipidemic effects of its flower extracts through integrated in vitro and in vivo models [19]. Furthermore, prior investigations by Ayachi et al. [20] demonstrated that the harvest season significantly influences the phytochemical composition of *A. henoniana* stems, thereby modulating their associated antioxidant and antimicrobial potencies. Despite these findings, the chemical and bioactive profiles of *A. henoniana* leaves remain largely unexplored. This study seeks to address this gap by providing a detailed evaluation of the leaf extracts alongside the floral components.

We are persuaded that as well adapted legume to pre‐Saharan conditions, *A. henoniana* is potentially producing secondary metabolites that are concomitantly useful for plant cell resistance to the arid climate conditions and as bioactive compounds. Therefore, we have undertaken a comprehensive phytochemical and pharmacological investigation of *A. henoniana* leaves and flowers to evaluate their bioactive potential.

The chemical profile (total phenolic and flavonoid contents, by spectrophotometer, phenolic composition by HPLC‐DAD assay, the antioxidant (anti‐DPPH assay), as well as the biological activities; cytotoxic (against MCF‐7 and HCT‐116 cancer lines), anti‐Alzheimer (anti‐acetylcholinesterase enzyme), and anti‐inflammatory (anti‐5‐lipoxygense enzyme). To provide mechanistic insights, in silico molecular docking and ADME (Absorption, Distribution, Metabolism, and Excretion) analyses were performed. Specifically, we targeted the Human Peroxidase (1PRX) structure, which has emerged as a critical multifunctional enzyme and a promising therapeutic target for managing oxidative stress‐related pathologies and oncogenesis.''

## 2 Results and Discussion

### 2.1 Extraction Yield, TPC, and TFC

According to the literature, no previous studies have been reported on the effect of organs and solvent polarity on the extraction yield, total polyphenol and flavonoids contents of *A. henoniana* organs (leaves and flowers). Regardless of solvent polarity, the highest extraction yield percentages were recorded for leaves (Table 1). The MeOH extract of leaves showed the highest yield value with 13.3%, accounting about 4.75 times higher than that of flower extract (2.8%) (Table 1). It seems that aerial parts of *A. henoniana* are mostly enriched in polar secondary metabolites soluble in methanol solvent. In fact, it was reported that the yield and extract composition tightly depended on the extraction process as well as solvent polarity [21, 22, 23].

**TABLE 1:** Yield (%) of organic extracts from leaves and flowers of *Anthyllis henoniana*.

	Leaves	Flowers
**Cyclohexane**	1.0	1.0
**Dichloromethane**	0.8	0.5
**Ethyl acetate**	0.4	0.5
**Methanol**	13.3	2.8

**FIGURE 1:**
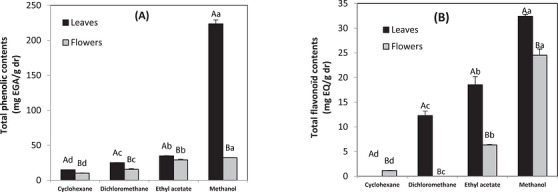
(A) Total phenolic and (B) flavonoid contents in organic extracts (cyclohexane; dichloromethane; ethyl acetate and methanol) from leaves and flowers of *Anthyllis henoniana*. Different capital letters indicate significant differences between organs; Different lowercase letters indicate significant differences between solvents. Data are reported as the means ± SE (*n* = 3) (*p* < 0.05).

The different *A. henoniana* extracts showed a modest total polyphenol content (TPC) ranging from 10.2 to 34.7 mg GAE/g dr. Exceptionally, the MeOH extract showed a high TPC with a value of 223.6 mg GAE/g dr (Figure 1A). Statistically, there was a significant difference (*P* ≤ 0.05) between organs and the used‐solvents extraction in terms of TPC. Therefore, we recorded about 7‐fold higher polyphenol contents in methanolic extract of leaves compared to the flowers of *A. henoniana* (Figure 1A). Polyphenols of leaves were mostly extracted with methanol, whereas flowers showed lower contents for all tested organic solvents (Figure 1A). Here, it seems that methanol polarity has the ability to extract a wide variety of active compounds compared to other solvents (Table 1). The total flavonoid contents (TFC) were proportionally increasing with solvent polarity (Figure 1B). The TFC of *A. henoniana* leaves exceeded by 25% that of flowers in methanolic extracts (Figure 1B).

Overall, the present findings demonstrated that the methanolic extracts of *Anthyllis henoniana* leaves and flowers are characterized by a significant enrichment in phenolic and flavonoid constituents. While Ayachi et al. [20] previously reported higher total phenolic (TPC) and flavonoid contents (TFC) than those observed in the current study, such discrepancies in secondary metabolite accumulation are likely attributable to fluctuations in edaphoclimatic conditions, including thermal stress, humidity, and the specific phenological stage at the time of harvest. Nevertheless, in other Mediterranean Fabaceae species, such as, *Sulla coronariam*, comparable TPC and TFC values have been reported, indicating that the phenolic profile of *A. henoniana* is consistent with plants adapted to similar constraints conditions [24]. Moreover, *Anthyllis vulneraria*, another Fabaceae species widely distributed in Mediterranean environments, was reported to contain a comparable TPC and TFC, particularly in aerial tissues, where these phytochemical constituents have been strongly correlated with pronounced radical scavenging activity [25].

### 2.2 HPLC Analysis of *A. henoniana Organ* Extracts

**FIGURE 2:**
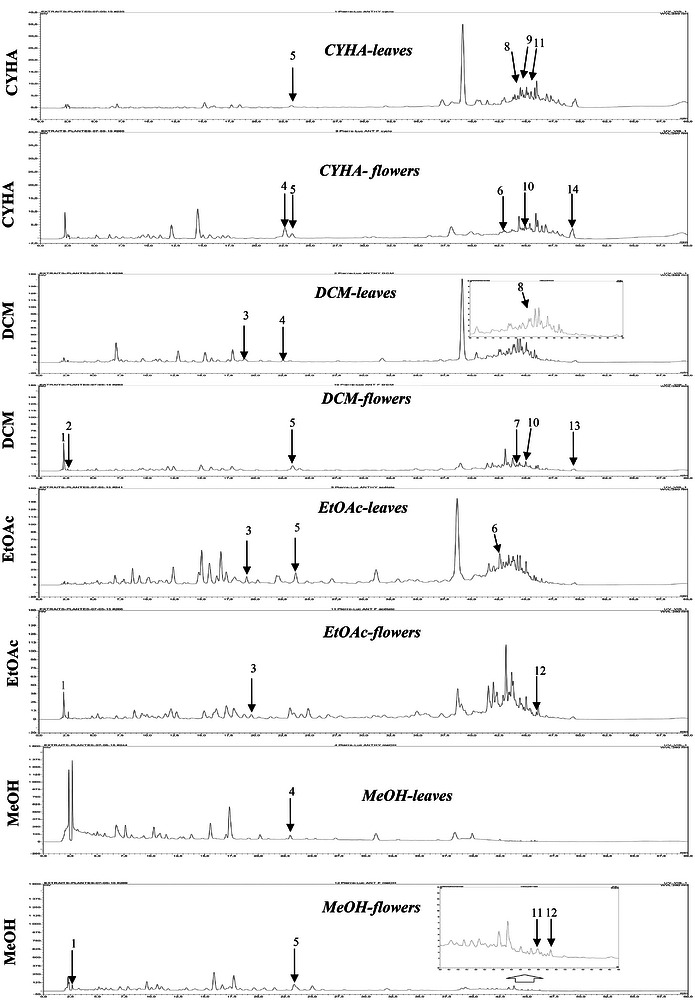
HPLC chromatograms of different organic extracts of *Anthyllis henoniana*. (CYHA: cyclohexane; DCM: dichloromethane; EtOAc: ethyl acetate; MeOH: methanol). (1): 3‐amino‐4‐hydroxybenzoic acid; (2): Gallic acid; (3): L‐tyrosine 7‐amido‐4‐methylcoumarin; (4): Rutin; (5): polydatin; (6): Butyl gallate; (7): Cardamonin; (8): Phenoxodiol; (9): Pinostilbene; (10): 3‐Benzyloxy‐4,5‐dihydroxy‐benzoic acid methyl ester; (11): Ethyl trans‐2‐hydroxycinnamate; (12): 4',5‐Dihydroxy‐7‐methoxyflavone; (13): 3,6,3‐Trimethoxyflavone; (14): 5‐Hydroxy‐3’‐methoxyflavone.

The identification of the phenolic compounds in the different extracts of *A. henoniana* organ (leaves and flowers) was determined using the HPLC‐DAD (Figure 2). In total, fourteen phenolic compounds were, tentatively, identified and quantified in all extracts (Table 2). Overall, the results indicated a marked variation in both qualitative and quantitative composition between the two plant organs. These compounds were distributed as follows; eight compounds in the leaves extracts, and twelve compounds in the flower's ones. While, five compounds were concomitantly found in leaf and flower extracts. These findings indicate a pronounced biochemical specialization among the different tissues of *A. henoniana*

**TABLE 2:** Phenolic compounds, tentatively, identified in the different extracts of *Anthyllis henoniana* leaves and flowers using HPLC‐DAD.

NO	Rt (min)	Compounds	Concentration (mg/g)								References
			Leaves				Flowers				
			CYHA	DCM	EtOAc	MeOH	CYHA	DCM	EtOAc	MeOH	
**1**	2.10	3‐Amino‐4‐hydroxybenzoic acid	—	—	—	—	—	0.45	0.38	—	[29]
**2**	3.48	Gallic acid	—	—	—	15.89	—	0.42	—	0.11	[30]
**3**	19.19	L‐Tyrosine 7‐amido‐4‐methylcoumarin	—	0.90	2.50	—	—	—	0.50	—	[31]
**4**	22.72	Rutin	—	0.93	—	—	1.61	—	—	—	[32]
**5**	23.37	Polydatin	0.67	—	—	—	0.59	3.83	—	2.48	[33]
**6**	42.97	Butyl gallate	—	—	8.5	—	0.07	—	—	—	[34]
**7**	44.17	Cardamonin	—	—	—	—	—	0.71	—	—	[35]
**8**	44.28	Phenoxodiol	0.05	1.12	7.18	—	—	—	—	—	[36]
**9**	44.64	Pinostilbene	0.64	—	0.55	—	—	—		—	[37]
**10**	44.96	3‐Benzyloxy‐4,5‐dihydroxy‐benzoic acid methyl ester	—	—	—	—	0.36	1.21	—	—	[38]
**11**	45.30	Ethyl trans‐2‐hydroxycinnamate	0.50	—	—	—	—	—	—	1.32	[39]
**12**	46.29	4',5‐Dihydroxy‐7‐methoxyflavone	—	—	—	—	—	—	1.93	1.50	[40]
**13**	47.99	3,6,3′‐Trimethoxyflavone	—	—	—	—	—	0.13	—	—	[41]
**14**	49.28	5‐Hydroxy‐3’‐methoxyflavone	—	—	—	—	1.00	—	—	—	[42]

Rt: retention time; ‐: not detected; CYHA: cyclohexane, DCM: dichloromethane; EtOAc: ethyl acetate; MeOH: methanol

The amounts of the identified compounds were ranging from 0.05 (phenoxodiol) to 15.89 mg/g (gallic acid) for leaves and from 0.07 (butyl gallate) to 3.83 mg/g (polydatin) for flower extracts. Polydatin was the only detectable compound in the four extracts (L‐CYHA, F‐CYHA, F‐EtOAc, and F‐MeOH), with a respective concentration of 0.67, 0.59, 3.83, and 2.48 mg/g. However, gallic acid, phenoxodiol and L‐tyrosine 7‐amido‐4‐methylcoumarin compounds were found in three extracts each. Within the leaves, gallic acid was identified as the predominant metabolite, reaching a concentration of 15.89 mg/g in the methanolic (MeOH) extract. It was followed by butyl gallate and phenoxodiol, which were detected at levels of 8.50 mg/g and 7.18 mg/g, respectively, in the ethyl acetate (EtOAc) extract. Furthermore, L‐tyrosine 7‐amido‐4‐methylcoumarin and phenoxodiol were detected at comparatively lower levels, not exceeding 2.5 mg/g in the leaf ethyl acetate (L‐EtOAc) and leaf dichloromethane (L‐DCM) extracts, respectively.

However, in the flower extracts, the highest concentration was recorded for polydatin in both dichloromethane (DCM) and methanolic (MeOH) fractions, with values of 3.83 mg/g and 2.48 mg/g, respectively. In addition, a second group of metabolites comprised intermediate‐abundance compounds, generally ranging from 1.21 to 1.93 mg/g. These included 4′,5‐dihydroxy‐7‐methoxyflavone, detected at 1.93 mg/g and 1.50 mg/g in the ethyl acetate (EtOAc) and methanolic (MeOH) extracts, respectively, as well as rutin, quantified at 1.61 mg/g in the CYHA extract. Ethyl trans‐2‐hydroxycinnamate was identified at 1.32 mg/g in the MeOH extract, while 3‐benzyloxy‐4,5‐dihydroxy‐benzoic acid methyl ester was detected at 1.21 mg/g in the corresponding extract.

While the leaves exhibited higher cumulative phenolic concentrations, particularly of phenolic acids such as gallic acid, suggesting a potentially stronger overall antioxidant capacity. In contrast, the flowers were characterized by a more diversified phytochemical profile, notably enriched in flavonoid and stilbene derivatives, reflecting a broader structural complexity of secondary metabolites. derivatives. The observed organ‐dependent metabolic partitioning is consistent with the phytochemical patterns previously reported for *Anthyllis vulneraria*, where HPLC–MS analyses revealed that leaf tissues were predominantly enriched in phenolic acids, whereas flower tissues generally exhibit higher levels and greater structural diversity of flavonoid derivatives. This tissue‐specific distribution supports the hypothesis that a recurrent biochemical differentiation between vegetative and reproductive organs may represent a conserved metabolic trait within the genus *Anthyllis* [26].

These differential metabolic patterns between leaves and flowers likely reflect the distinct physiological roles and metabolic specialization of each organ. Numerous studies have reported that secondary metabolite accumulation is highly tissue‐dependent, with concentrations and compound diversity varying according to organ function, developmental stage, and exposure to environmental pressures such as light, herbivory, and oxidative stress [27, 28].

### 2.3 Anti‐DPPH Activity

**FIGURE 3:**
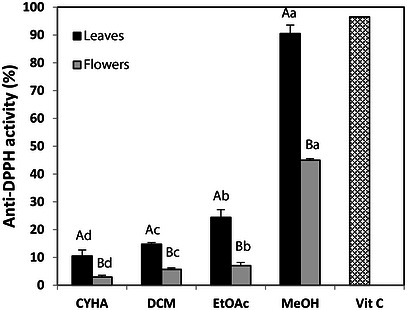
Evaluation of anti‐DPPH activity in organic extracts (CYHA: cyclohexane; DCM: dichloromethane; EtOAc: ethyl acetate and MeOH: methanol) from leaves and flowers of *Anthyllis henoniana* assayed at 50 µg/mL. Different capital letters indicate significant differences between organs; Different lowercase letters indicate significant differences between solvents. Data are reported as the means ± SE (*n* = 3), (*p* < 0.05). Vit C: vitamin C.

The antioxidant activity, based on DPPH free radicals scavenging capacity, was at least two‐fold higher in leaves than in flowers of *A. henoniana* for all tested organic extracts (Figure 3).

It could be stated that the antioxidant activity increased with increasing solvent polarity. For instance, the highest DPPH free radical inhibition percentages were recorded for the MeOH extract, with around 90 and 45% for leaves and flowers, respectively (Figure 3). Concordantly, the best IC_50_ of about 7.7 µg/mL was calculated for the L‐MeOH extract (Table 3). Obtained data have shown that antioxidant activity was highly correlated with TPC (*r* = 0.92), as well as with TFC (*r* = 0.91) (Table 4). Hence, the better antioxidant activity in the two organs may be related to its higher phenolic compounds (Figure 1A). In fact, many works have argued for a close relationship between polyphenol levels and antioxidant activity [43, 44]. The highest antioxidant activity obtained for the methanolic fraction suggested that the detected compounds by HPLC (Figure 2) are acting in an additive and/or synergistic manner to stabilize or deactivate free radicals. A statistically significant difference (*p* ≤ 0.05) was observed between the methanolic extracts of leaves and flowers. This variation in DPPH radical scavenging capacity is likely due to the difference in the richness of chemical compounds present in the extracts from these two aerial parts (Table 2). For instance, in the leaf MeOH extracts, there was a high correlation between L‐tyrosine 7‐amido‐4‐methylcoumarin and the anti‐DPPH activity (*r* = 0.90) (Table 4). The present antioxidant capacity of the *Anthyllis henoniana* extracts was higher compared to the results found by Ayachi et al. [45] where the best antioxidant potential of the methanolic extracts of *Anthyllis sericea* was recorded in the stem harvested in spring. However, certain identified compounds with well‐documented intrinsic antioxidant activity, such as rutin and phenoxodiol, did not show a significant statistical correlation with the measured antioxidant activity. This apparent discrepancy may be explained by the fact that the overall antioxidant potential of complex plant extracts is not solely determined by the intrinsic redox properties of individual constituents but also by their relative abundance, their partitioning across different solvent fractions, and potential synergistic or antagonistic interactions among co‐occurring metabolites [46].

**TABLE 3:** IC_50_ (µg/mL) values of the anti‐DPPH, anti‐LOX, and anti‐AChE activities of organic extracts (cyclohexane; dichloromethane; ethyl acetate; methanol) of leaves and flowers from *Anthyllis henoniana*.

	Anti‐DPPH IC_50_ (µg/mL)		Anti‐5‐LOX IC_50_ (µg/mL)		Anti‐AChE IC_50_ (µg/mL)	
	Leaves	Flowers	Leaves	Flowers	Leaves	Flowers
**Cyclohexane**	>50	>50	>50	15.2 ± 7.3	>50	NA
**Dichloromethane**	>50	>50	47.0 ± 3.4	NA	>50	NA
**Ethyl acetate**	>50	>50	NA	>50	>50	NA
**Methanol**	7.7 ± 3.1	>50	42.0 ± 1.7	>50	>50	50.0 ± 0.5
**Ascorbic acid**	2.0±0.0		—			—
**Nordihydroguaretic acid**	—		2.2 ± 0.1			—
**Galanthamine**					6.5±0.2	

NA: not active

**TABLE 4:** Pearson's correlation matrix^a^.

Variables	TPC	TFC	DPPH	5‐Lox	AChE	MCF‐7	HCT‐116	C1	C2	C3	C4	C5	C6	C7	C8	C9	C10	C11	C12	C13	C14
**TPC**	1																				
**TFC**	0.75	1																			
**DPPH**	0.92	0.91	1																		
**5‐Lox**	0.23	0.05	0.16	1																	
**AChE**	0.99	0.75	0.92	0.31	1																
**HCT‐116**	0.31	0.44	0.28	0.09	0.39	0.82	1														
**C3**	0.99	0.70	0.90	0.25	0.98	−0.18	0.31	−0.24	−0.16	1											
**C4**	−0.27	0.14	−0.01	0.48	−0.21	−0.34	−0.22	−0.40	−0.27	−0.30	1										
**C6**	−0.08	0.22	−0.01	−0.46	−0.11	0.45	0.49	−0.22	−0.14	−0.06	−0.27	−0.16	1								
**C7**	−0.22	−0.35	−0.30	0.69	−0.19	−0.19	−0.26	−0.22	−0.14	−0.16	0.47	−0.15	−0.14	1							
**C8**	−0.08	0.22	−0.01	−0.46	−0.11	0.45	0.49	−0.22	−0.14	−0.06	−0.27	−0.16	1.00	−0.14	1						
**C14**	−0.22	−0.35	−0.30	0.69	−0.19	−0.19	−0.26	−0.22	−0.14	−0.16	0.47	−0.15	−0.14	1.00	−0.14	−0.22	0.11	−0.24	−0.21	−0.143	1

^a^ Only statistically significant correlations are highlighted. (3): L‐tyrosine 7‐amido‐4‐methylcoumarin; (4): rutin; (6): Butyl gallate; (7): Cardamonin; (8): Phenoxodiol; (14): 5‐hydroxy‐3’‐methoxyflavone.

### 2.4 Cytotoxic Activity

**FIGURE 4:**
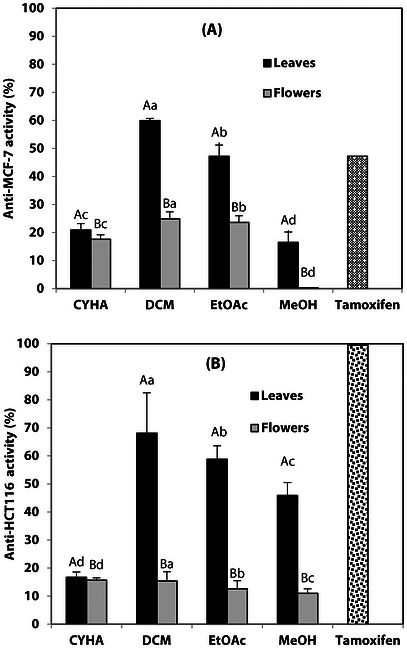
Cytotoxic activity of *Anthyllis henoniana* extracts against MCF‐7 (A) and HCT‐116 (B) cell lines. Different capital letters indicate significant differences between organs; Different lowercase letters indicate significant differences between solvents.

The cytotoxic activity of the various extracts of *A. henoniana* was evaluated, for the first time, against two cell lines, MCF‐7 and HTC‐116 (Figure 4A, B).

The highest inhibition percentages against MCF‐7 cell lines of about 60% and 48% were recorded for the DCM and EtOAc of leaf extracts. However, the inhibition percentage of the same extracts from flowers organ did not exceed 25%. Likewise, the other extracts (CYHA and MeOH) of both organs were not ideally efficient against MCF‐7 cells. Their inhibition percentages do not outstrip 30%. In addition, the highest percentage of inhibition against HCT‐116 was assigned to the leaf DCM extract (68.1%), followed by the EtOAc (58.8%) and MeOH (45.9%) extracts. The other organic extracts, whether for leaves or flowers, were unable to effectively inhibit the HCT‐116 cell line. Their inhibition percentages did not exceed 20%.

Statistical analysis revealed a weak negative correlation between total bioactive content (TPC and TFC) and the MCF‐7 cell line (*r* = −0.19 and *r* = −0.12, respectively). Conversely, a moderate positive correlation was observed with the HCT‐116 cell line (*r* = 0.31 for TPC and *r* = 0.45 for TFC). A previous study of Aguero et al. [47] showed that phenoxodiol displayed a potent antiproliferative effect against the HCT‐116 cell lines. Moreover, the phenoxodiol was also reported as an apoptosis inducer in chemoresistant cancer cells, such as breast cancer [48]. In the current findings, there was a moderate correlation between the cytotoxic activity (anti‐MCF‐7 and anti‐HCT‐116) and the phenoxodiol compound, with r‐values of 0.45 and 0.49, respectively (Table 4). The cytotoxic screening conducted in the present study serves as a preliminary evaluation of the *A. henoniana* therapeutic potential. Although specific extracts demonstrated significant growth inhibition against the MCF‐7 and HCT‐116 adenocarcinoma cell lines, these data neither establish tumor selectivity nor elucidate the underlying molecular mechanisms of action.

### 2.5 Anti‐Acetylcholinesterase (AChE) Activity

**FIGURE 5:**
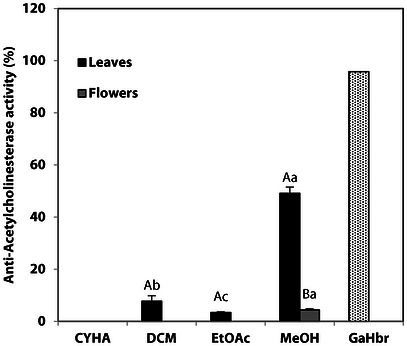
Anti‐acetylcholinesterase activity of *Anthyllis henoniana* extracts. A capital letters mean the difference between species; A lowercase letters mean difference between solvents. Different letters on the histograms mean a significant difference according to the Tukey test at *P* ≤ 0.05. GaHbr: Galantamine Hydrobromide.

The anti‐AChE activity of *A. henoniana* leaf and flower extracts has not been studied previously. Except for the MeOH‐leaf extract, all the extracts of both organs showed a very low or no AChE inhibitory activity (Figure 5). Both leaves and flowers IC_50_ values were higher than 50 mg/mL for all tested organic extracts (Table 3). The MeOH‐leaf extract showing the highest activity against the ACHE enzyme (49.1%) was the most enriched in total polyphenols (Figure 1A). This finding was consolidated by a linear correlation statistic, which indicated a positive value between TPC and AChE activity (*r* = 0.99), as well as between TFC and AChE activity (*r* = 0.75) (Table 4). Moreover, a strong positive correlation was found between L‐tyrosine 7‐amido‐4‐methylcoumarin (coumarins) and AChE activity (*r* = 0.98) (Table 4). According to the study of Alipour et al. [49], 7‐hydroxycoumarin derivatives revealed a significative effect against the AChE enzyme.

### 2.6 Anti‐5‐Lipoxygenase (5‐LOX) Activity

**FIGURE 6:**
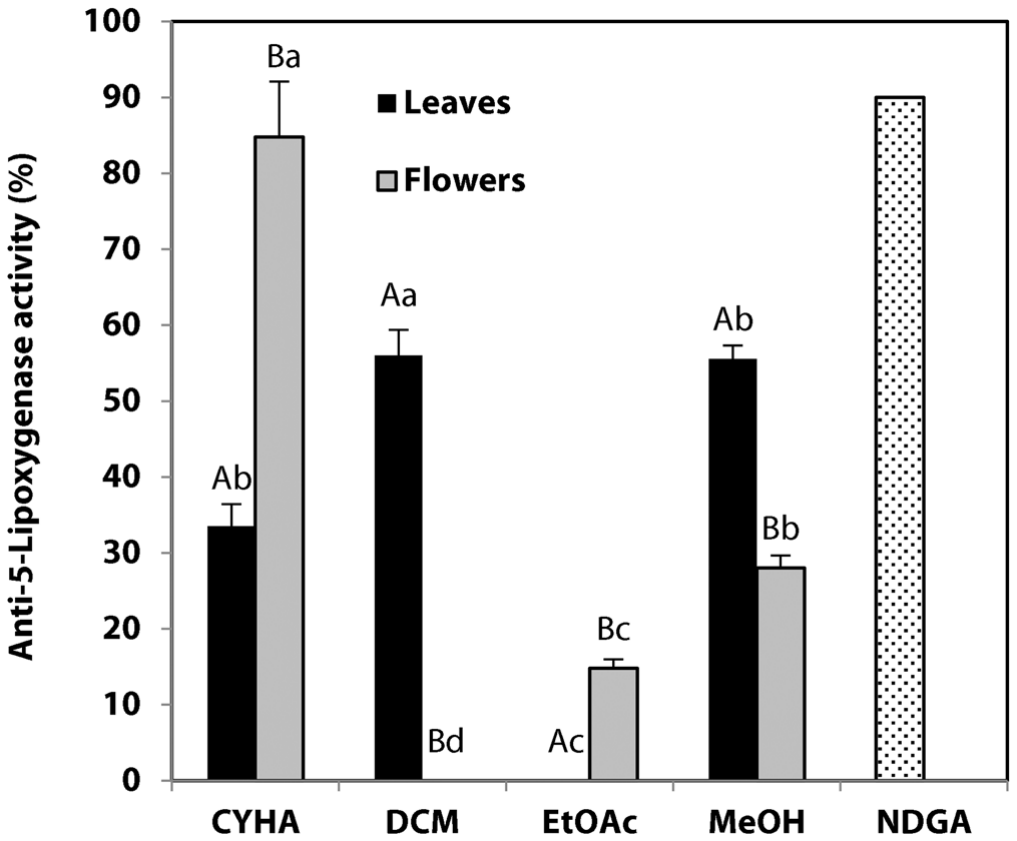
Changes in the anti‐5‐lipoxygenase activity of *Anthyllis henoniana* extracts. A capital letters mean the difference between species; a lowercase letters mean difference between solvents. Different letters on the histograms mean a significant difference according to the Tukey test at *P* ≤ 0.05. NDGA: Nordihydroguaiaretic acid.

The 5‐lipoxygenase inhibitory activity of *A. henoniana* leaf and flower extracts was evaluated in comparison to the standard nordihydroguaiaretic acid, and the results were shown in Figure 6 as the inhibition percentage. The tested extracts showed a percentage‐values ranging from 0% to 56.02 % for leaves and from 0% to 84.79 % for flowers against the 5‐LOX enzyme. The Leaf‐DCM and Leaf‐MeOH extracts exhibited a moderate anti‐5‐LOX activity of 56.02% and 55.57 %, which correspond to IC_50_ values of 44.7 and 44.2 µg/mL, respectively (Table 3). However, the flower‐CYHA extract showed the highest activity against the 5‐LOX of 84.79 %, with IC_50_ of 15.20 µg/mL (Table 3).

In the current study, a good correlation was found between the cardamonin and the anti‐5‐LOX, as well as between the 5‐hydroxy‐3’‐methoxyflavone and the anti‐5‐LOX with *r‐value* of 0.69 and 0.69, respectively (Table 4). The cardamonin exhibits a variety of pharmacological activities including anti‐inflammatory effects [50]. In addition, several studies confirmed that the 5‐hydroxy‐3’‐methoxyflavone, which was detected in the CYHA‐flowers extract, was involved in the anti‐inflammatory reaction [51, 52].

### 2.7 In silico Analysis

**FIGURE 7:**
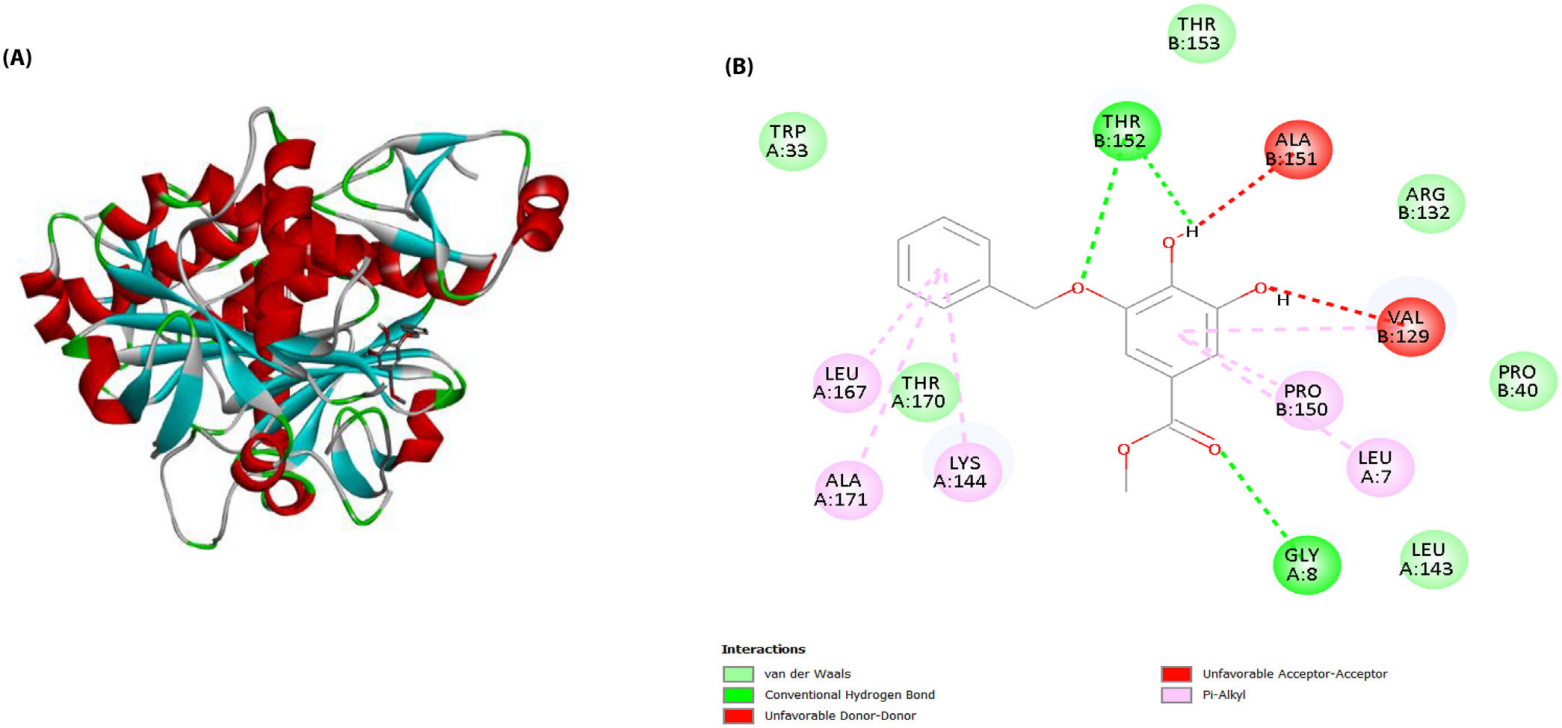
Binding interaction of the 3‐Benzyloxy‐4,5‐dihydroxy‐benzoic acid methyl ester compound with the antioxidant target protein (1PRX) based on the binding energy generated by PyRx program: (a) 3D diagram of ligand–protein binding pose; (b) 2D illustration of ligand–protein interactions.

Virtual screening was conducted to generate hypotheses regarding the potential affinity of each compound to selected target proteins: 1PRX as an antioxidant protein model and 2IUJ as an anti‐inflammatory protein model. Blind docking performed using PyRx software revealed that all the tested compounds were successfully docking into the target proteins (Table 5). All the identified compounds in the *Anthyllis henoniana* extracts were docked within the active pocket of the targeted enzymes. According to the virtual screening results (Table 6), the 3‐benzyloxy‐4,5‐dihydroxy‐benzoic acid methyl ester and the L‐tyrosine‐7‐amido‐4‐methylcoumarin exhibited the best binding affinity with the 1PRX protein model. However, the rutin and the 5‐hydroxy‐3’‐methoxyflavone displayed the best binding affinity with the 2IUJ protein model. Based on the obtained results (Table 6), it could be stated that the phytocompounds 3‐benzyloxy‐4,5‐dihydroxy‐benzoic acid methyl ester and the L‐tyrosine7‐amido‐4‐methylcoumarin compound can bind with the antioxidant enzyme (1PRX) with the highest docking scores of −7.7 and −7.6, respectively, which were better than the DPPH radical (‐7.2 kcal/mol). The binding interaction between 1PRX and 3‐benzyloxy‐4,5‐dihydroxy‐benzoic acid methyl ester revealed the formation of two conventional hydrogen bonds with THR152 (4.21 Å and 2.99 Å) and GLY8 (3.74 Å), involving the oxygen atom of the ligand (Figure 7A). In addition, multiple π‐alkyl interactions were observed with LEU167 (6.07 Å), ALA171 (6.07 Å), LYS144 (5.54 Å), LEU7 (6.61 Å), and PRO150 (4.80 Å) (Figure 7B). These interactions collectively contribute to the stable accommodation of the ligand within the active pocket of the antioxidant enzyme [53].

**TABLE 5:** Docking binding energies (kcal/mol) and interaction of the ligands into the active site of antioxidant (1PRX) and anti‐inflammatory (2IUJ).

NO	Phytocompounds	(1PRX) Binding affinity (Kcal/mol)	(2IU) Binding affinity (Kcal/mol)
**1**	3‐Amino‐4‐hydroxybenzoic acid	−5.6	−6.0
**2**	Gallic acid	−5.8	−6.0
**3**	L‐Tyrosine‐7‐amido‐4‐methylcoumarin	−7.6	−8.6
**4**	Rutin	−7.2	−9.2
**5**	Polydatin	−7.4	−8.4
**6**	Butyl gallate	−6.0	−6.4
**7**	Cardamomin	−6.8	−7.9
**8**	Phenoxodiol	−6.8	−7.8
**9**	Pinostilbene	−6.5	−7.3
**10**	3‐Benzyloxy‐4,5‐dihydroxy‐benzoic acid methyl ester	−7.7	−7.8
**11**	Ethyl trans‐2‐hydroxycinnamate	−5.6	−6.2
**12**	4',5‐Dihydroxy‐7‐methoxyflavone	−6.8	−8.5
**13**	3,6,3′‐Trimethoxyflavone	−6.8	−7.5
**14**	5‐Hydroxy‐3’‐methoxyflavone	−7.3	−9.3
	DPPH radical (reference)	−7.2	—
	Nordihydroguaiaretic acid (reference)	—	−7.6

**TABLE 6:** Drug likeness results of the selected compounds from *Anthyllis henoniana* leaves and flowers extracts.

		H‐Bond									Violation				
Molecule	MW	Acceptors	Donors	Fraction Csp3	Rotatable bonds	MR	TPSA (A^2^)	Log P	ESOL Log S	ESOL class	Lipinski	Ghose	Veber	Egan	Muegge
3‐Amino‐4‐hydroxybenzoic acid	150.11	3	3	0.00	1	36.34	83.55	−0.02	−1.42	Very soluble	0	3	0	0	1
Gallic acid	168.10	5	4	0.00	1	37.14	97.99	0.09	−1.63	Very soluble	0	2	0	0	1
L‐Tyrosine‐7‐amido‐4‐methylcoumarin	324.25	5	3	0.16	5	79.94	105.56	1.54	−3.35	Soluble	0	1	0	0	0
Rutin	590.36	16	10	0.44	6	120.67	269.43	−3.89	−3.17	Soluble	3	2	1	1	3
Polydatin	464.97	8	8	0.30	5	162.80	139.84	−0.36	−2.80	Soluble	1	1	0	1	1
Butyl gallate	215.14	5	3	0.36	5	48.06	86.99	0.98	−2.64	Soluble	0	1	0	0	0
Cardamomin	258.18	4	2	0.06	4	62.06	66.76	1.81	−3.83	Soluble	0	0	0	0	0
Phenoxodiol	230.17	3	2	0.07	1	56.90	49.69	1.34	−2.85	Soluble	0	0	0	0	0
Pinostilbene	213.17	2	1	0.07	1	53.64	29.46	2.30	−3.89	Soluble	0	1	0	0	0
3‐Benzyloxy‐4,5‐dihydroxy‐benzoic acid methyl ester	262.17	5	2	0.13	5	59.23	75.99	1.42	−3.17	Soluble	0	0	0	0	0
Ethyl trans‐2‐hydroxycinnamate	181.12	3	1	0.18	4	41.46	46.53	1.34	−2.74	Soluble	0	1	0	0	1
4',5‐Dihydroxy‐7‐methoxyflavone	274.18	5	2	0.06	2	67.18	79.90	1.65	−4.08	Moderately soluble	0	0	0	0	0
3,6,3′‐trimethoxyflavone	298.21	5	0	0.28	4	66.83	53.99	1.48	−3.59	Soluble	0	0	0	0	0
5‐hydroxy‐3’‐methoxyflavone	257.18	4	1	0.06	2	63.99	59.67	2.07	−4.58	Moderately soluble	0	0	0	0	0

Abbreviations: MW: molecular weight, MR: molar refractivity

Furthermore, compounds such as cardamomin and pinostilbene exhibited moderate binding affinities (−6.8 and −6.5 kcal/mol, respectively), whereas smaller phenolic molecules showed comparatively weaker interactions, as exemplified by 3‐amino‐4‐hydroxybenzoic acid (−5.6 kcal/mol). This trend suggests a clear structure‐dependent stabilization of the ligand–protein complex, where molecular size, functional group distribution, and hydrophobic interactions play a key role in binding efficiency

**FIGURE 8:**
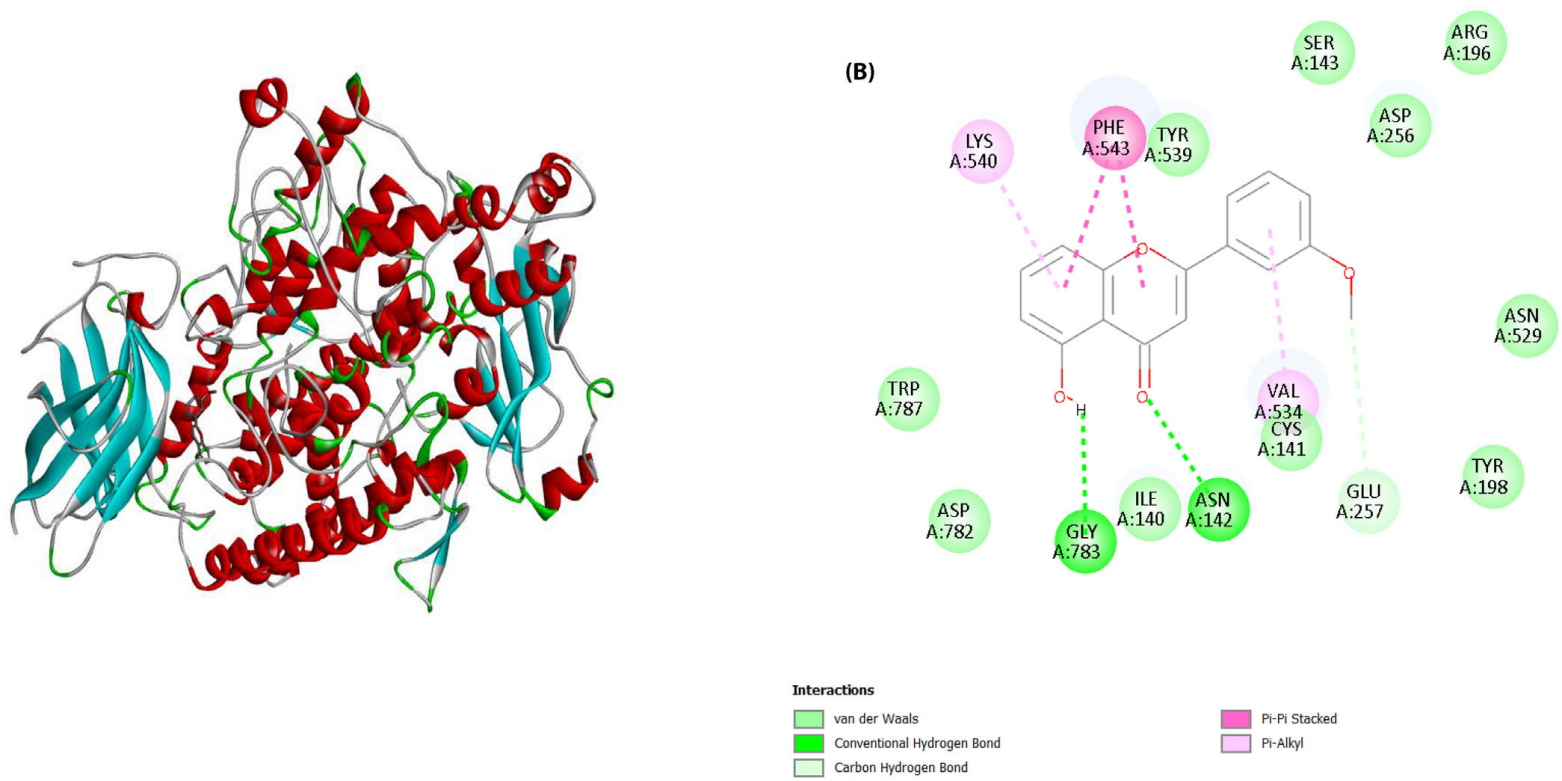
Binding interaction of the 5‐hydroxy‐3’‐methoxyflavone compound with the 5‐lipoxygenase target protein (2IUJ) based on the binding energy generated by PyRx program: (a) 3D diagram of ligand–protein binding pose; (b) 2D illustration of ligand–protein interactions.

The enzyme (PDB: 2IUJ) was selected as a lipoxygenase protein target since the different extract of *A*. *henoniana* showed an inhibitory activity against this enzyme (Table 6). We found that the 5‐hydroxy‐3’‐methoxyflavone strongly interacted with the active site residues of the 2IUJ protein, showing a binding energy of ‐9.3 kcal/mol. This value was higher compared to the reference (nordihydroguaiaretic acid) with a binding energy of ‐6.7 kcal/mol. Rutin, L‐tyrosine‐7‐amido‐4‐methylcoumarin, 4',5‐dihydroxy‐7‐methoxyflavone, and polydatin showed a respective binding energy value of ‐9.2, ‐8.6, 8.5, and ‐8.4 kcal/mol (Table 6). The Figure 8 showed that the 5‐hydroxy‐3’‐methoxyflavone was attached to two conventional hydrogen bonds, namely, GLY783 (2.71 Å), and ASN142 (3.78 Å). It also demonstrated a pi alkyl interaction with LYS540 (6.09 Å), and VAL534 (5.44 Å), as well as a pi–pi stacked interactions with PHE543 (4.95 Å and 4.77 Å) (Figure 8). These interactions are known to improve ligand stabilization within the catalytic pocket. Furthermore, an intermediate and lower binding were found for some compounds such as, cardamomin (−7.9), phenoxodiol (−7.8), and 3‐amino‐4‐hydroxybenzoic acid (−6.0).

### 2.8 ADME Analysis of Selected Phytoconstituents with Best Docking Score

Compounds from *Anthyllis henoniana* extracts were selected in terms of their maximum binding affinity (best docking score), and were then subjected to ADME analysis by using SwissADME online software. This online tool provides information about the pharmacokinetics, physicochemical properties and drug likeness attributes of the selected compounds with best docking score [54]. It should be stated that there are no available reports regarding the ADME analysis of *Anthyllis henoniana* in the literature.

The qualification of each selected compound as an oral drug was conducted according to the criteria of Lipinski, Ghose, Veber, Egan, and Muegge (Table 6). Lipinski's rule of five is as follows: molecular weight < 500 Da, <5 hydrogen bond donors, <10 hydrogen bond acceptors, and log p < 5 [55]. While Ghose uses four parameters to qualify a molecule as drug: lows: 160 Da ≤ molecular weight ≤ 480 Da, −0.4≤ log P ≤ 5.6, 40 ≤ molar refractivity ≤ 130, and 20 ≤ total atom number ≤ 70 [56]. Veber bases drug likeness on two parameters: topological polar surface area (TPSA) < 140 A2 and number of rotatable bonds <10 [57]. Egan considers two criteria: TPSA <132 A2 and −1 ≤ log P ≤ 6 [58]. Muegge establishes drug likeness as follows: 200 Da ≤ molecular weight ≤ 400 Da, −2 ≤ log P ≤ 5, TPSA ≤ 150 A2, <5 hydrogen bond donors, <10 hydrogen bond acceptors, <15 rotatable bonds, <7 rings, and at least four carbon atoms and one heteroatom [59]. Solubility was measured using the ESOL model, where a log *S*‐value ≤ −6 indicates a poorly soluble compound, while a log S value ≤ −10 indicates an insoluble compound. Additionally, a compound with bioavailability score between 0.55 and 0.85, and 0.25 ≤ Csp3 (degree of flexibility) score ≤ 1 is qualified as orally bioavailable [60]. According to Lipinski's and criteria, all molecules in the *A. henoniana* leaves and flowers extracts may be suggested as oral drug candidates, except for rutin and polydatin compounds. According to Ghose's criteria, almost all molecules could be as drug candidates except for 3‐amino‐4‐hydroxybenzoic acid, gallic acid, rutin, and polydatin. In addition, almost the half of the selected compounds were classified as drug candidates when using the Veber and Egan criteria.

The filter process using Muegge's criteria showed that the 3‐amino‐4‐hydroxybenzoic acid, gallic acid, the rutin, polydatin, and the ethyl trans‐2‐hydroxycinnamate were not qualified as drug candidate.

The bioavailability and Csp3 score revealed that all the selected compounds were orally bioavailable, except for the rutin compound. Furthermore, compounds with no more than one violation are promising drug candidates. The Figures 7 and 8 described the bioavailability radar of selected phytoconstituents from *Anthyllis henoniana* extracts.

#### 2.8.1 Analysis of Pharmacokinetic Properties

For each selected compound, the pharmacokinetic parameters (Table 7) were estimated for gastrointestinal absorption (GI), blood–brain barrier (BBB) permeability, and skin permeability (log Kp). A lower skin permeability of the molecule was indicated by a more negative log Kp. The interaction between the molecule and specific proteins, such as cytochromes P450 (CYPs) and permeability glycoprotein (P‐gp), are another factor that underlies pharmacokinetics. The P‐gp is essential for active efflux in the direction of the biological membrane, whereas CYPs are proteins involved in the biotransformation of drugs. The score for synthetic accessibility goes from 1 (extremely easy) to 10 (extremely difficult). Most of the identified phenolic compounds, particularly gallic acid, pinostilbene, and ethyl trans‐2‐hydroxycinnamate, exhibited favorable pharmacokinetic profiles, suggesting their potential for good absorption, distribution, and overall drug‐likeness. These compounds demonstrated high gastrointestinal absorption, no predicted inhibition of the major CYP450 isoforms, suggesting a low potential for drug–drug interactions, together with favorable bioavailability scores ranging from 0.55 to 0.56. They also exhibited low synthetic accessibility values, indicating relative ease of synthesis and/or compatibility with natural occurrence. It could be noted that this favorable ADME profiles suggest strong potential for further development as orally bioavailable bioactive ingredients. On the other hand, compounds 5‐hydroxy‐3’‐methoxyflavone and 4’,5‐dihydroxy‐7‐methoxyflavone had more favorable physicochemical profiles with moderate molecular weight and balanced lipophilicity, pointing to a better potential for passive absorption. In contrast, rutin showed a markedly low bioavailability score (0.17) and poor GI absorption, which may explain its weak correlation with the observed antioxidant activity despite being identified by HPLC. Interestingly, phenoxodiol and 5‐Hydroxy‐3’‐methoxyflavone were the only compounds predicted to cross the blood‐brain barrier, opening perspectives for neuroprotective applications. Overall, these in silico ADME results support the promising potential of the major phenolic compounds from *A. henoniana* aerial parts for further development as natural bioactive ingredients.

**TABLE 7:** Pharmacokinetic results of the selected compounds from *Anthyllis henoniana* extracts.

					Cyp Inhibitor						Alerts		
Molecule	Bioavailability Score	GI absorption	BBB permeant	P‐gp substrate	CYP1A2	CYP2C19	CYP2C9	CYP2D6	CYP3A4	Log Kp (cm/s)	PAINS	BRENK	Synthetic accessibility
3‐Amino‐4‐hydroxybenzoic acid	0.56	High	No	No	No	No	No	No	No	−6.86	0	2	1.00
Gallic acid	0.56	High	No	No	No	No	No	No	No	−6.83	1	1	1.16
L‐Tyrosine7‐amido‐4‐methylcoumarin	0.55	High	No	No	No	No	No	No	No	−6.74	0	1	2.81
Rutin	0.17	Low	No	Yes	No	No	No	No	No	−10.14	1	1	5.74
Polydatin	0.55	Low	No	No	No	No	No	No	No	−7.85	0	1	4.24
Butyl gallate	0.55	High	No	No	No	No	No	No	No	−5.90	1	2	1.59
Cardamomin	0.55	High	No	No	No	No	No	No	No	−5.39	0	1	2.23
Phenoxodiol	0.55	High	Yes	Yes	No	No	No	No	No	−6.13	0	0	3.72
Pinostilbene	0.55	High	Yes	No	No	No	No	No	No	−4.90	0	1	1.72
3‐Benzyloxy‐4,5‐dihydroxy‐benzoic acid methyl ester	0.55	High	No	No	No	No	No	No	No	−6.10	1	1	1.97
Ethyl trans‐2‐hydroxycinnamate	0.55	High	No	No	No	No	No	No	No	−5.47	0	1	1.90
4',5‐Dihydroxy‐7‐methoxyflavone	0.55	High	No	No	Yes	No	No	No	No	−5.59	0	0	2.71
3,6,3‐Trimethoxyflavone	0.55	High	No	No	Yes	Yes	No	No	No	−6.11	0	0	3.28
5‐Hydroxy‐3’‐methoxyflavone	0.55	High	Yes	No	No	No	No	No	No	−4.84	0	0	2.60

### 2.9 PCA and Correlation Coefficients Analysis

**FIGURE 9:**
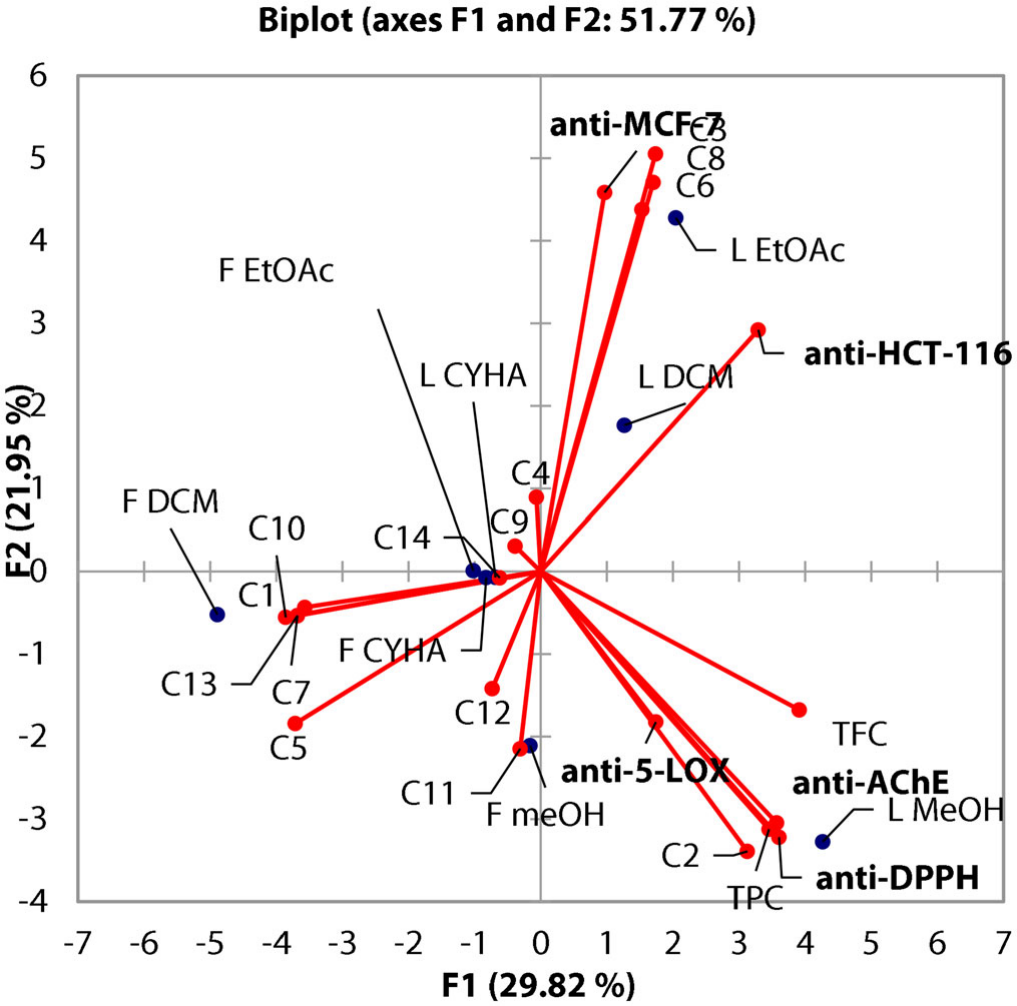
Principal component analysis (PCA) to elucidate the relationships between chemical composition, phenolic compounds, and the biological activities of *Anthyllis henoniana* extracts. L: Leaf; F: Flowers.

The antioxidant and the biological activities data of *A. henoniana* organ extracts were analyzed using PCA. The structuring of accessions showed 51.77% of the total variation (Figure 9). The axes were retained since they expressed 28.45% (F1) and 20.28% (F2) percentages. Simultaneously, the PCA loading plot clearly showed that the principal components correlated with the original variables, and that there was a correlation between the assayed activities and the TPC and TFC. As shown in Figure 9, The first principal component (F1) effectively characterized a phytochemical polarity gradient, segregating highly polar extracts from their non‐polar counterparts. The methanolic leaf extracts (L‐MeOH) were distinctly localized on the positive axis of F1, demonstrating robust positive correlations with antioxidant capacity (anti‐DPPH), neuroprotective potential (anti‐AChE), and total bioactive content (TPC and TFC). These associations were supported by high factor loadings of 0.72, 0.72, 0.69, and 0.79, respectively, indicating that the polar constituents of the leaves are the primary drivers of these biological activities. This clustering confirmed the well‐established relationship between phenolic compounds and antioxidant potential, as polyphenols are recognized for their ability to donate electrons or hydrogen atoms and neutralize free radicals.

Interestingly, the compound C2 (gallic acid) is closely aligned with this cluster in the PCA biplot, suggesting that this metabolite serves as a primary determinant of the observed antioxidant capacity [61]. On the other hand, the extract L‐EtOAc is located in the upper‐right quadrant, showing a strong association with the cytotoxic activity against MCF‐7 and HCT‐116 cancer cell lines (Figure 9). The localization of compounds C3 (L‐tyrosine 7‐amido‐4‐methylcoumarin), C6 (butyl gallate), and C8 (phenoxodiol) within this quadrant suggests that the semi‐polar metabolites concentrated in the ethyl acetate (EtOAc) fraction contribute substantially to the observed antiproliferative effects against the MCF‐7 and HCT‐116 adenocarcinoma cell lines. Multivariate analysis further substantiated this association, as the variables for anti‐MCF‐7, anti‐HCT‐116, and C3 exhibited moderate‐to‐strong positive correlations with the F2 axis, supported by robust factor loadings of 0.79, 0.50, and 0.87, respectively (Table 8).

**TABLE 8:** Correlations between variables and factors.

	F1	F2	F3
**TPC**	0.69	−0.54	−0.36
**TFC**	0.79	−0.29	−0.38
**DPPH**	0.72	−0.55	−0.37
**5‐LOX**	0.35	−0.31	0.77
**AChE**	0.72	−0.52	−0.31
**MCF‐7**	0.19	0.79	−0.10
**HCT‐116**	0.66	0.50	−0.22
**C1**	−0.72	−0.07	−0.43
**C2**	0.63	−0.58	−0.32
**C3**	0.35	0.87	−0.29
**C4**	−0.01	0.15	0.79
**C5**	−0.75	−0.32	−0.34
**C6**	0.31	0.75	−0.35
**C7**	−0.74	−0.09	−0.53
**C8**	0.34	0.81	−0.33
**C9**	−0.08	0.05	0.22
**C10**	−0.77	−0.10	−0.31
**C11**	−0.06	−0.37	0.10
**C12**	−0.15	−0.24	0.01
**C13**	−0.74	−0.09	−0.53
**C14**	−0.12	−0.01	0.74

The third axis (F3) showed a strong correlation with factor loading of 0.77 (Table 8). This activity appears correlated with F‐MeOH and the two compounds (C11; ethyl trans‐2‐hydroxycinnamate, and C12; 4',5‐dihydroxy‐7‐methoxyflavone), suggesting that these metabolites may contribute to the anti‐inflammatory mechanisms via inhibition of the lipoxygenase pathway

Overall, the PCA demonstrated that solvent polarity exerts a definitive influence on both the phytochemical architecture and the resulting pharmacological profile of *Anthyllis henoniana*, underscoring the criticality of optimized extraction strategies in natural product research. Furthermore, this multivariate approach facilitates the identification of specific metabolites, most notably gallic acid and polydatin as key bioactive markers. These findings suggest that such compounds may serve as standardized chemical fingerprints for the quality control and therapeutic standardization of *A. henoniana* extracts in future pharmaceutical applications [62].

## 3 Conclusions

The organic extracts of *Anthyllis henoniana* exhibited a different chemical and bioactive pattern, reflecting a specific phytochemical profiling depending on solvent polarity and plant organ. Definitely, *A. henoniana* leaf richness in polar phenolic and flavonoid constituents was associated with a strong antioxidant and cytotoxic activities. These results highlight the potential of polar leaf extracts for pharmaceutical development targeting oxidative stress‐related disorders and cancer. Additionally, the leaves displayed relatively higher acetylcholinesterase inhibitory activity, suggesting possible neuroprotective applications. Conversely, *A. henoniana* flowers accumulated nonpolar metabolites exhibiting pronounced anti‐5‐lipoxygenase activity. These findings warrant further targeted investigation to validate their potential application in the development of novel anti‐inflammatory therapeutic formulations.

The in vitro assays were combined to in silico bioinformatics‐based drug discovery approaches. Blind molecular docking revealed that the selected chemical compounds exhibited a strongest binding affinity with target protein model indicating a promising pharmacokinetic and drug‐likeness properties.

Overall, these findings suggest that *A. henoniana* has significant pharmacological versatility, with leaf‐derived polar compounds and flower‐derived nonpolar metabolites demonstrating promising in vitro antioxidant, anticancer, and anti‐inflammatory activities. However, the current study is inherently limited by its short experimental duration and in vitro scope. Future investigations should prioritize in vivo assessments of endogenous antioxidant enzyme activities and the modulation of pro‐inflammatory cytokines to better delineate the therapeutic efficacy of *A. henoniana* extracts. Furthermore, to validate our in silico findings, subsequent research should confirm the regulation of key target proteins—specifically 1PRX and 2IUJ—at both the transcriptional (gene expression) and translational (protein) levels. Such an integrative approach will be vital for substantiating the pharmacological relevance of these secondary metabolites in complex biological systems.

## 4 Experimental Section

### 4.1 Chemicals Used

All chemicals used were of analytical reagent grade. All reagents were purchased from Sigma, Aldrich (France): acetic acid, acetonitrile, CYHA, DCM, Dulbecco's modified eagle medium, dimethyl sulfoxide, doxorubicin, Folin‐Ciocalteu reagent (2 N), gallic acid, HCl, KH_2_PO_4_, MeOH, MTT, NaOH, Roswell Park Memorial Institute, 4‐nitrophenyl‐β‐D‐glucuronic acid, Na_2_CO_3_ and 15‐LOX.

### 4.2 Sample Collection

*A. henoniana* Coss. Ex Batt. samples were collected in the South of Tunisia in the mountains of Beni khedache delegation (Médenine, Tunisia) (Latitude: 33°15'42.5“N N, Longitude: 10°01'26.5”E). Specimen identification was assisted by Prof. Mohamed Debouba and colleagues from the environmental sciences department at the Higher Institute of Applied Biology of Medenine. A voucher specimen (Ahe 022) was deposited at the research unit of the High Institute of Applied Biology (Medenine, Tunisia). The collected *A. henoniana* species were divided into leaves and flowers and then air‐dried on the shadow, until weight stability (three weeks) and then dried to a fine powder.

### 4.3 Plant Extraction

The dried flowers and leaves of *A. henoniana* were fractionated with the following organic solvents: cyclohexane (CYHA), dichloromethane (DCM) and methanol (MeOH). The extraction was performed using a cold maceration process. A total of 100 g of powder was successively extracted with 1 L of each solvent during 2 h with using a magnetic agitator. After each extraction, the extract was filtered through filter paper (Whatman N° 2), and the solvent was removed using a rotary vacuum evaporator (IKA, RV 10 auto V, Germany) under pressure at 35C [63]. After solvent evaporation, yields were determined, and dry residues were used for subsequent assays. The yield extraction was determined as follows:

Yield(%)=(m/M)×100, with m: weight of dry residue (g); M: weight of plant material (g).

### 4.4 Phytochemical and Antioxidant Analysis

#### 4.4.1 Determination of the Total Phenolic Content (TPC)

The total polyphenol contents were determined according to the Folin‐Ciocalteu method, slightly modified by Bekir et al. [64]. Briefly, in 96‐well microplates, 20 µL of the selected extract (0.5 mg/mL) was mixed with 100 µL of Folin–Ciocalteu reagent (0.2 N), and 100 µL of sodium carbonate (75 %). The mixture was gently shaken and kept in the dark for 25 min. The absorbance of the extracts was measured in using a microplate reader (Multiskan Go, F1‐01620, Thermo Fisher Scientific) at 765 nm. A standard calibration curve was obtained using gallic acid (0–115 mg/L). Results were reported as mg equivalent gallic acid per g of dry residue (mg GAE)/g dr).

#### 4.4.2 Determination of the Total Flavonoids Content (TFC)

A slightly modified version of the spectrophotometric method was used to determine the flavonoids contents of samples [65]. Briefly, an aliquot (100 µL) of the extract (0.5 mg/mL) was added with 100 µL of AlCl_3_ in MeOH (2%). After 15 min of incubation at room temperature, the absorbance was measured at 410 nm against a reagent blank (MeOH) and diluted plant extract without AlCl_3_. Total flavonoids content was expressed as mg equivalent quercetin per g of dry residue (mg EQ/g dr).

#### 4.4.3 High Performance Liquid Chromatographic Analysis (HPLC‐DAD)

Analytical HPLC‐DAD (Thermo Fisher Scientific, USA) analysis was conducted for the chemical compounds’ identification in the *A. henoniana* organic extracts. The separation was performed at room temperature (20 to 25°C) on Reverse Phase‐C18 column at elution of 1.2 mL/min flow rate. The mobile phase (pH = 2.65) included (A) acidified water and (B) water/acetonitrile (20:80 v/v). The program proceeded as the following gradient: 35 min from 0.1% to 30% B, 5 min from 30% to 50% B, 5 min from 50 to 99.9% B, and 15 min from 99.9 to 0.1% B. The *A. henoniana* samples were prepared at 20 mg/mL and detected at 280 nm. The identification of detected metabolites was performed by comparing their retention times (RT) and UV–Vis spectral profiles against authentic analytical standards. Furthermore, these data were cross‐referenced with established phytochemical databases and previously reported profiles of *Anthyllis* species and related taxa within the Fabaceae family to ensure taxonomic and phytochemical consistency [66].

#### 4.4.4 Anti‐DPPH Scavenging (Antioxidant Activity)

Antioxidant scavenging activity was measured by the slightly modified spectrophotometric method of Blois [67]. A volume of 20 µL of plant extract (0.5 mg/ mL) was added to 180 µL DPPH solution (0.2 mM) and the mixture was allowed to stand. The microplate reader was used to measure the absorbance at 520 nm, the wavelength of maximum DPPH absorbance. The A sample, after an incubation period of 25 min at room temperature (15–20°C), was measured. Ascorbic acid (0.5 mg/mL) was used as positive control (standard).

### 4.5 Biological Activities

#### 4.5.1 Cytotoxic Activity

The cytotoxic activity of *A. henoniana* extracts against cancer cell lines. The human colorectal carcinoma cell line HCT‐116 (RRID:CVCL_0291) and the human breast adenocarcinoma cell line MCF‐7 (RRID:CVCL_0031) were obtained from the American Type Culture Collection (ATCC, Manassas, VA, USA). The cytotoxic activity was estimated by the 3‐ (4,5‐dimethylthiazol‐2‐yl)‐2,5‐diphenyltetrazolium bromide (MTT) assay as described by Sahpazidou et al. [68], with minor modifications. Cells were distributed into 96‐well plates at 3×10^4^ cells/well in 100 µL, and then 100 µL of culture medium; DMEM for MCF‐7 or RPMI for HCT‐116 containing samples at different concentrations were added. Metabolically active cells are able to convert the dye to water‐insoluble dark blue formazan by reductive cleavage of the tetrazolium ring. The extracts were re‐solubilized in the DMSO followed by dilution in the buffer whereby the DMSO does not exceed 1%. Tamoxifen was used as a positive control (0.2 µg/mL). The A_blank_ was measured without extract. The cells activity inhibition percentage was calculated as:

$$\%\mathrm{inhibition}=100\times \left(A_{\mathrm{blank}}-A_{\mathrm{sample}}\right)/A_{\mathrm{blank}}$$

#### 4.5.2 Anti‐acetylcholinesterase Activity (AChE)

Acetylcholinesterase (AChE) inhibitory activity was measured using Ellman's method, as modified by Owokotomo et al. [69]. Briefly, 50 µL of sodium phosphate buffer (0.1 mM, pH = 7.5), 125 µL of DTNB (3 mM), 25 µL of plant extract (0.5 mg/mL), and 25 µL of acetylthiocholine solution (493.2 U), obtained from *Electrophorus electricus* (Sigma‐Aldrich), were mixed and incubated for 15 min at 25°C. After that, 25 µL of ACTHi (15 mM) was added. Then the final solution was incubated for 25 min at 25°C and the absorbance was measured at 421 nm. The A_blank_ was measured without extract.

Galantamine Hydrobromide (GaHb) was used as positive reference. The enzyme activity inhibition percentage was calculated as:

$$\%\mathrm{inhibition}=100\times \left(A_{\mathrm{blank}}-A_{\mathrm{sample}}\right)/A_{\mathrm{blank}})$$

#### 4.5.3 Anti‐15‐lipoxygenase Activity (5‐LOX)

Human 5‐lipoxygenase (5‐LOX) catalyzes the oxidation of the linoleic acid to a conjugated diene. The 5‐LOX enzyme was derived from soybean (Sigma‐Aldrich). The obtained conjugated diene was quantified to evaluate the anti‐5‐LOX activity as described by Bekir et al. [61]. In Brief, 20 µL of extract (0.5 mg/ mL) was mixed with 170 µL of Na_3_PO_4_ buffer (0.1 mM, pH 7.4), 60 µL of linoleic acid (3,5 mM), and 20 µL of 5‐LOX solution (soybean 500 U). However, the blank has not contained the substrate, replaced by the buffer solution. The standard LOX inhibitor nordihydroguaiaretic acid (NDGA) was used as a positive reference. The mixture was incubated at 25°C for 10 min, and the absorbance was determined at 234 nm. The inhibition of enzyme activity was calculated as follows:

$$\%\mathrm{inhibition}=100\times \left(A_{\mathrm{blank}}-A_{\mathrm{sample}}\right)/A_{\mathrm{blank}}$$

#### 4.5.4 Molecular Docking

*In‐silico* studies on the phytocompounds from *A. henoniana* organ extracts, obtained from the HPLC‐DAD analysis, was performed to understand the binding interaction of the identified compounds within the active pocket of the targeted enzymes; antioxidant and lipoxygenase. PyRx used as the docking tool, while Discovery Studio (v24, 2023) was used for visualization and preparation of proteins (water and ligand molecules removing). The structures were taken from the RCSB Protein Data Bank (https://www.rcsb.org/): antioxidant (PDB code: 1PRX, Resolution: 2.0 Ă) [70], and 5‐lipoxygenase (PDB code: 2UIJ, Resolution: 2.40 Ă) [71]. The soybean lipoxygenase‐B (PDB: 2IUJ) was selected as a molecular target due to its structural homology with human 5‐lipoxygenase (5‐LOX), the rate‐limiting enzyme in the biosynthesis of leukotrienes, which are potent mediators of inflammatory responses. Concurrently, human peroxiredoxin (PDB: 1PRX) was chosen as a target to elucidate the mechanistic basis of the observed antioxidant activity; this enzyme plays a critical role in cellular homeostasis by neutralizing reactive oxygen species (ROS) and mitigating oxidative stress‐induced damage. The ligands were downloaded from PubChem database (https://pubchem.ncbi.nlm.nih.gov/). The results highlighted the compounds showing the most favorable binding affinities with these enzymes.

#### 4.5.5 ADMET Studies

The SwissADME (http://www.swissadme.ch, accessed date 23th October 2025) online tools was used for evaluating the pharmacokinetic parameters including; gastrointestinal (GI) absorption, log of skin permeability (log Kp), permeability glycoprotein (Pgp) substrate, blood brain barrier penetration (BBB), cytochrome P450 (CYP450) enzymes‐CYP2D6, CYP3A4, CYP2C9, CYP2C19 inhibitors. This tool was used to determine the ADME (absorption, distribution, metabolism, and excretion) properties of compounds as estimate and indicators of pharmacokinetics [72].

#### 4.5.6 Statistical Analysis

All experiments were conducted four times and the statistical difference was analyzed using the Statistical Package for the Social Sciences (SPSS) 26.0 software. Statistical differences between the solvents used in the study were estimated using Tukey's test. The determination of the relationship between TPC and biological activities was assessed by the coefficient of determination (R^2^). The principal component analysis (PCA) was also performed using XLSTAT (version 5.03) to visualize the difference between all the parameters.

## Author Contributions

Experiments were done by SJ, under the direction of the coauthors. Article writing was done by SJ with the help of RR. JB followed up the experimental part. RR performed the statistical analysis and in silico study. MD and JB validated the experiments, proofread, and refined the article to make it ready for publication. All authors have read and approved the finalized manuscript.

## Conflicts of Interest

The authors confirm that they have no conflicts of interest with respect to the work described in this article.

## Data Availability

The data that support the findings of this study are available from the corresponding author upon reasonable request.
